# The association between Histone 3 Lysine 27 Trimethylation (H3K27me3) and prostate cancer: relationship with clinicopathological parameters

**DOI:** 10.1186/1471-2407-14-994

**Published:** 2014-12-23

**Authors:** Marjolaine Ngollo, Andre Lebert, Aslihan Dagdemir, Gaelle Judes, Seher Karsli-Ceppioglu, Marine Daures, Jean-Louis Kemeny, Frederique Penault-Llorca, Jean-Paul Boiteux, Yves-Jean Bignon, Laurent Guy, Dominique Bernard-Gallon

**Affiliations:** Department of Oncogenetics, Centre Jean Perrin, CBRV, 28 place Henri Dunant, BP 38, 63001 Clermont-Ferrand, France; EA 4677 “ERTICA”, University of Auvergne, 28 place Henri Dunant, BP 38, 63001 Clermont-Ferrand, France; Institut Pascal UMR 6602 CNRS/UBP, University Blaise Pascal, 24 Avenue des Landais, Aubiere, France; Department of Anatomo-pathology, Gabriel Montpied Hospital, 58 rue Montalembert, 63001 Clermont-Ferrand, France; Department of Urology, Gabriel Montpied Hospital, 58 rue Montalembert, 63001 Clermont-Ferrand, France

**Keywords:** Chromatin immunoprecipitation, Epigenetic, Prostate cancer, Histone modifications, Trimethylation

## Abstract

**Background:**

It is well established that genetic and epigenetic alterations are common events in prostate cancer, which may lead to aberrant expression of critical genes. The importance of epigenetic mechanisms in prostate cancer carcinogenesis is increasingly evident. In this study, the focus will be on histone modifications and the primary objectives are to map H3K27me3 marks and quantify *RAR beta 2, ER alpha, SRC3, RGMA, PGR,* and *EZH2* gene expressions in prostate cancer tissues compared to normal tissues. In addition, a data analysis was made in connection with the clinicopathological parameters.

**Methods:**

71 normal specimens and 66 cancer prostate tissues were randomly selected in order to assess the proportion of the repressive H3K27me3 mark and gene expression. H3K27me3 level was evaluated by ChIP-qPCR and mRNA expression using RT-qPCR between prostate cancer and normal tissues. Subsequently, western-blotting was performed for protein detection. The analysis of variance (ANOVA) was performed, and Tukey’s test was used to correct for multiple comparisons (p-value threshold of 0.05). The principal component analysis (PCA) and discriminant factorial analysis (DFA) were used to explore the association between H3K27me3 level and clinicopathological parameters.

**Results:**

The study demonstrated that H3K27me3 level was significantly enriched at the *RAR beta 2, ER alpha, PGR,* and *RGMA* promoter regions in prostate cancer tissues compared to normal tissues. After stratification by clinicopathological parameters, the H3K27me3 level was positively correlated with Gleason score, PSA levels and clinical stages for *RAR beta 2, ER alpha, PGR,* and *RGMA*. High H3K27me3 mark was significantly associated with decreased *RAR beta 2, ER alpha, PGR* and *RGMA* gene expressions in prostate cancer sample compared to the normal one. Moreover, the results showed that mRNA level of *EZH2, AR* and *SRC3* are upregulated in prostate cancer compared to normal prostate tissues and this correlates positively with Gleason score, PSA levels and clinical stages. Obviously, these observations were confirmed by protein level using western-blot.

**Conclusions:**

This data clearly demonstrated that H3K27me3 level correlated with aggressive tumor features. Also this study revealed that reverse correlation of *RAR beta 2, ER alpha, PGR,* and *RGMA* expressions with *EZH2*, *SRC3,* and *AR* expressions in prostate cancer tissues suggests that these genes are the target of EZH2. Therefore, all therapeutic strategies leading to histone demethylation with epigenetic drugs such as histone methyltransferase inhibitor may be relevant treatments against prostate cancer.

## Background

Prostate cancer is the most frequently diagnosed cancer in men in the western world and second leading cause of cancer death in males worldwide [1]. In 2011, prostate cancer represented 71,200 new cases and 8,700 deaths in France. Prostate cancer like many other malignancies arises from progressive genetic and epigenetic alterations [2]. Tumorigenesis and progression of prostate cancer result from the accumulation of genetic and epigenetic alterations. Epigenetic modifications include several different phenomena, such as DNA methylation [3], histone modifications [4], and microRNAs (miR) regulation [5]. Basically, epigenetics regulate gene expression and play an important role in carcinogenesis [6].

In this study, the focus is put on histone modifications and their role in prostate cancer progression. Studies have shown that histone modifications contribute to the onset and progression of prostate cancer [7]. Common histone modifications leading to gene silencing in prostate cancer include histone H3 lysine 9 methylation (H3K9me3), histone deacetylation, and polycomb-based histone H3 lysine 27 trimethylation (H3K27me3) [8]. Polycomb-mediated H3K27me3 has been shown to play critical role in diverse biological processes, such as development, stem cell maintenance, transcriptional silencing of homeotic gene, and in early steps of X-chromosome inactivation in women [9].

So far, it is important to understand the role of histone modifications in the control of gene transcription. H3K27me3 is catalyzed by the polycomb enhancer of zeste homolog 2 (EZH2), the catalytic core protein of the polycomb repressor complex 2 (PRC2). This histone methyltransferase is well known in initiating target gene silencing by promoting H3K27me3 leading to the chromatin condensation [10]. Many authors have demonstrated that overexpression of *EZH2* was strongly associated with progression and invasion of prostate cancer [11]. In addition, some studies showed that *EZH2* is upregulated by aberrant expression of MYC transcription factor and microRNA [12]. Clearly, MYC promotes *EZH2* expression by repressing the expression of *miR-26a* and *miR-26b*, which might be a negative regulator of *EZH2* [13].

Kondo et al. (2008) found that up to 5% of promoters were enriched with H3K27me3 and showed none or low DNA methylation in their promoters. This data establish EZH2-mediated H3K27me3 like a mechanism of gene silencing in cancer potentially independent of DNA methylation [8].

In fact, to study histone methylation, a selection of six genes involved in prostate cancer was made, including *RAR beta 2, ER alpha, PGR, RGMA, EZH2,* and *SRC3*.

The retinoic acid receptor (RAR) is a transcription factor that regulates transcription of set genes involved in biological processes such as apoptosis, proliferation and cellular differentiation. Of course, *RAR beta 2* is one of the genes involved in aberrant methylation in human prostate cancer [14, 15]. Previous studies reported that the methylated promoter region of *RAR beta 2* in prostate cancer cell lines (LNCaP and PC3) was associated with both hypoacetylation and hypermethylation of histone H3 [9]. However, few studies knew about the mechanisms underlying the involvement of histone methylation upon the silencing of *RAR beta 2* expression in tumor cells, until Moisson et al. (2013) confirmed that DNA hypermethylation cannot explain by itself the epigenetic repression of *RAR beta 2* gene [15].

Truly, prostate cancer accelerates the osteoblastic differentiation during the process of metastasis interacting with bone mophogenetic proteins (BMPs) [16]. Recently, repulsive guidance molecule A (RGMA) a GPI-linked membrane protein has been identified as co-receptor of bone morphogenetic proteins (BMPs) [17]. Kondo et al. (2008) demonstrated that, *RGMA* expression was significantly lower in cancer tissues than in normal ones. However, the underlying mechanisms are not well understood yet [8].

Also, it is well established that androgen receptor (AR) plays a critical role in prostate cancer cell proliferation, survival, and differentiation. But, some reports also dealt with the potential implication of other two steroid hormone nuclear receptors, estrogen receptor (ER) alpha and progesterone receptor (PGR) in prostatic carcinogenesis [18]. In any case, in the normal human prostate, immunohistochemical studies have revealed a stromal localization of ER alpha and PGR, and less or no ER alpha expression was detected in malignant prostate epithelium in various prostates [19]. *PGR* is the major ER alpha responsive gene, its expression is not detected in malignant prostatic epithelium [18].

Finally, steroid receptor coactivator 3 (SRC3) is a member of the p160 family of coactivators for nuclear hormone receptors including the androgen receptor. For instance, previous studies have shown that *SRC3* is overexpressed in prostate cancer cells and its overexpression correlates with prostate cancer proliferation and is inversely correlated with apoptosis [20].

The aim of this study was to assess the association between H3K27me3 level and prostate cancer risk and the correlation of H3K27me3 on *EZH2*, *RAR beta 2, ER alpha, PGR,* and *RGMA* promoters with clinicopathological variables including Gleason score, PSA levels and clinical stages. Therefore, normalizing H3K27me3 by targeting inhibition of EZH2 seems to become a potential new method for cancer therapy.

## Results

### H3K27me3 correlated positively with clinicopathological parameters

**Figure 1:**
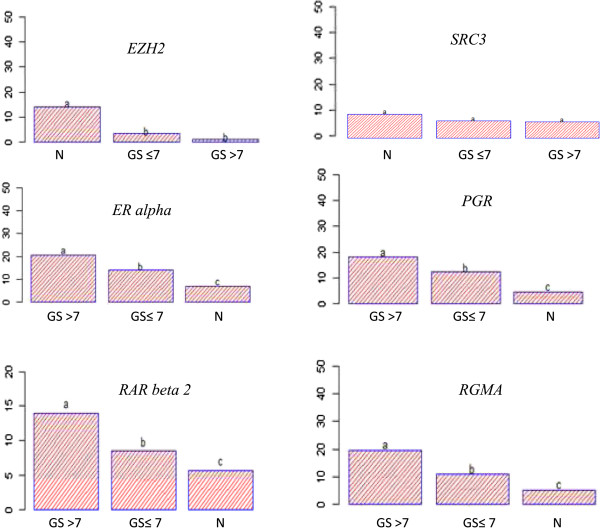
**Assessment of H3K27me3 marks in normal and tumoral tissues using ChIP-qPCR.** ChIP analysis indicates the change of H3K27me3 marks at six gene loci in prostate cancer tissues. H3K27me3 level on *EZH2* was found to be lower in prostate cancer tissues (n = 32) versus normal tissues (n = 33). Contrariwise, the H3K27me3 level of *RAR beta 2, PGR*, *ER alpha* and *RGMA* was significantly higher in tumoral tissues that in normal tissues. The H3K27me3 level on SRC3 in cancer tissue did not reach statistical significance compared in normal tissue. The data is expressed as % of input. Analysis of variance, followed by a Tukey multiple comparison test, was used for statistical analysis. The statistical significant between groups was indicated by letters “a”, “b” and “c”. (N = normal; GS = Gleason score).

**Figure 2:**
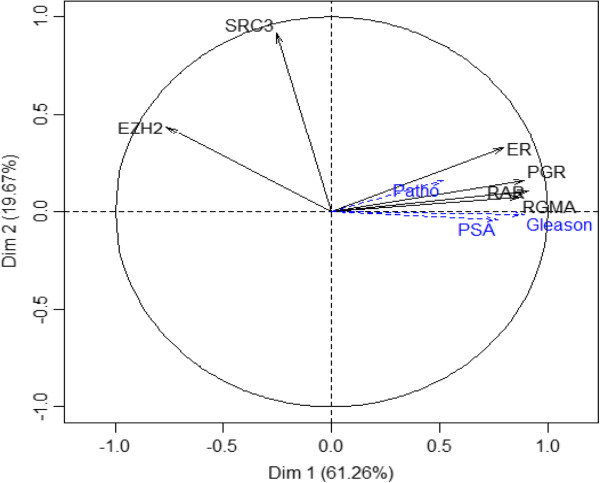
**Principal component analysis (PCA) carried out to explore the relationships between H3K27 levels and clinicopathological parameters.** H3K27me3 level was evaluated by PCA analysis. H3K27me3 levels on *ER alpha*, *RAR beta 2*, *RGMA* and *PGR* genes correlated positively with PSA level (PSA), clinical stage (patho) and Gleason score (Gleason).

Firstly, using ChIP assay, we demonstrated that H3K27me3 occupancy at *RAR beta 2, ER alpha*, *PGR*, and *RGMA* promoters is increased in tumoral tissue compared to normal tissue unlike to *EZH2* promoter. We did not find obvious H3K27me3 modification signals at the *SRC3* promoter (Figure 1). These results suggest that EZH2 regulates *RAR beta 2, ER alpha*, *PGR* and *RGMA*. On the contrary, *SRC3* expression would be regulated in an H3K27me3-independent manner. The H3K27me3 level in tumoral and normal tissues was then explored by principal component analysis (PCA) to assess the relationship between Gleason score, PSA levels, and clinical stages. Additionally, the extracted PCA factors were used to examine the ability to discriminate between patients with cancer and healthy patients. PCA showed that the total variance explained by the first principal component (Dim1) of PCA was 61.26% whereas the second principal component (Dim2) of PCA explained nearly 19.67% of the total variance. As shown in Figure 2, on the first principal component, a clear discrimination can be seen between *EZH2* gene and *RAR beta 2, ER alpha*, *PGR* and *RGMA* genes. *SRC3* is orthogonal to the horizontal axis and therefore did not participate to PCA analysis. PCA results demonstrated a close relationship between H3K27me3 level and Gleason score, PSA levels and clinical stages. This means that, patients with a high proportion of H3K27me3 marks on *RAR beta 2, ER alpha*, *PGR* and *RGMA* genes have a high Gleason score and advanced clinical stage.

**Figure 3:**
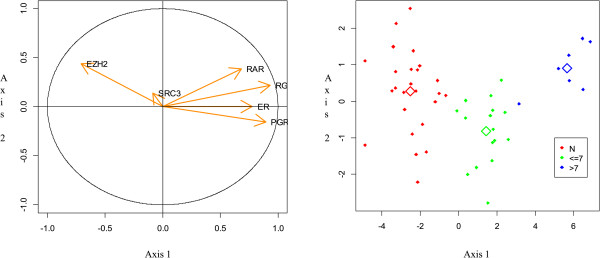
**Factorial Discriminant Analysis (FAD) carried out to describe the variables that discriminate the three groups of patients.** RAR beta 2, ER alpha, RGMA and PGR variables conflict to EZH2. Variable SRC3 being away from the circle, it wears a low information (left panel). The graphical representation of patients was used to identify homogeneous groups within the population from the point of view of variable studied (H3K27me3). Red plots represent normal patients (N), green plots represent patients with Gleason score ≤ 7 (<=7) and blue plots represent patients with Gleason score >7 (>7) (right panel).

In a second step, discriminant factorial analysis (DFA) was applied to the PCA data that take into account the information contained in the raw data sets. DFA has allowed classifying patients according to different groups. For that purpose, patients in the study cohort were divided into two groups, 75% of patients have formed learning group and 25% validation group (Table 1). It has been noted that although Gleason score, PSA levels and clinical stages variables displayed high correlation, only the Gleason score was able to distinguish the three groups (N, GS ≤ 7 and GS > 7) with a significant percentage of around 99%. All patients are correctly classified, only 1 out of 48 patients was misclassified (Table 2). On the validation group, patients were 100% correctly classified (Table 3). The graphical representation of patients was described in Figure 3 and a clear discrimination between N, GS ≤ 7 and GS > 7 groups were observed. The same process was applied to clinical stages data. Regarding T1 clinical stage group, correct classification amounting to 80% was observed. Nevertheless, T2 clinical stage group was only 67% correctly classified. The T3 clinical stage group was not analyzed because we have only one patient in this group. None of the three groups (T1, T2 and T3 clinical stage) was 100% correctly classified, the classification rate was not satisfactory. However, with PSA levels, it was not possible to predict the membership of different groups established relative to H3K27me3 level.

**Table 1:** **Distribution of patients in two groups**

	Learning group	Validation group	Total
Normal	25	8	33
Gleason score ≤7	16	3	19
Gleason score > 7	7	5	12
Total	48	16	64

75% of patients allowed establishing learning database and 25% validation database.

**Table 2:** **Classification of patients with different groups (normal, Gleason score ≤ 7 and Gleason score > 7)**

Observed patients	Predicted patients	Normal	Gleason score ≤ 7	Gleason score > 7	% of correct classification
Normal (n = 25)		**25**	0	0	100
Score ≤7 (n = 16)		0	**16**	0	100
Score >7 (n = 7)		0	1	**6**	85.8
Total		25	17	6	

This table represents a learning group and represents 75% of patients. The percentage of correctly classified patients corresponds to the ratio of the number of patients well classified by the total number of patients (n). It is observed that there is one individual of Gleason score >7 group is misclassified. The number in bold corresponded to patients well classified.

**Table 3:** **Classification of patients with different groups (normal, Gleason score ≤ 7 and Gleason score > 7)**

Observed patients	Predicted patients	Normal	Gleason score ≤ 7	Gleason score > 7	% of correct classification
Normal (n = 8)		**8**	0	0	100
Score ≤7 (n = 3)		0	**3**	0	100
Score >7 (n = 5)		0	0	**5**	100
Total		8	3	5	

This table represents a validation group and represents 25% of the patients. The percentage of correctly classified patients corresponds to the ratio of the number of patients well classified by the total number of patients (n). We observed that all patients are well classified.

### Gene expression in prostate tissues

**Figure 4:**
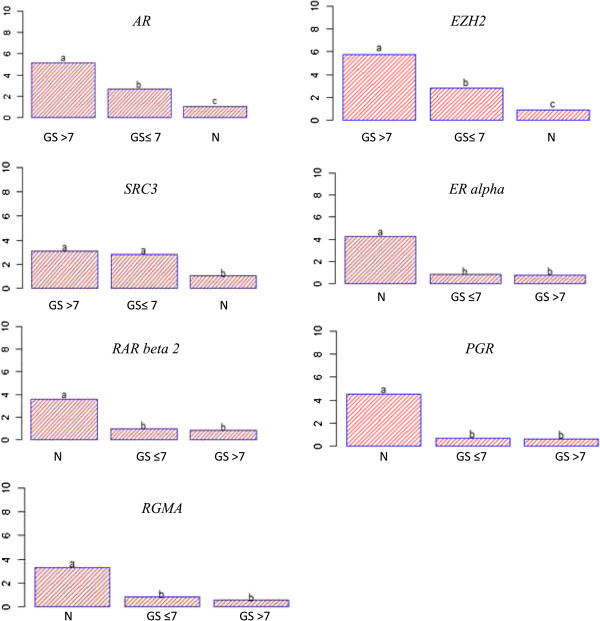
***EZH2*** **,** ***AR*** **and** ***SRC3*** **is upregulated in prostate cancer and inversely correlated with** ***RAR beta 2*** **,** ***ER alpha*** **,** ***PGR*** **and** ***RGMA*** **.** mRNA expression of seven genes were measured using RT-qPCR in normal tissues (n = 38) and cancerous tissues (n = 34). 18S RNA was used as an internal control in PCR reactions. All genes that have high H3K27me3 levels in their promoter are consistent with the low mRNA expression levels. In contrast, genes that have low H3K27me3 levels are consistent with high mRNA expression levels. Analysis of variance, followed by a Tukey multiple comparison test, was used for statistical analysis. The statistical significant between groups was indicated by letters “a”, “b” and “c”. (N = normal; GS = Gleason score).

**Figure 5:**
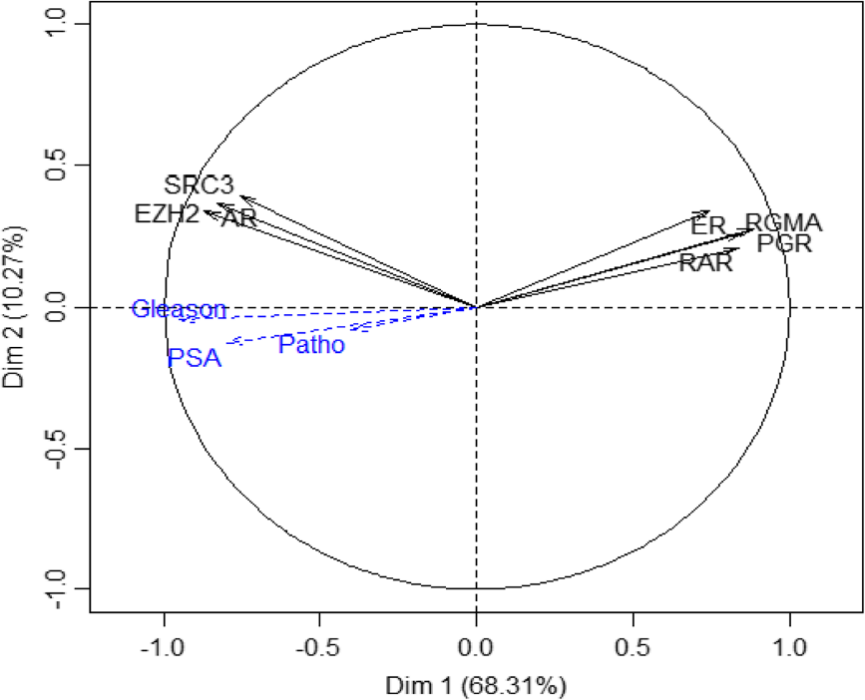
**Principal component analysis (PCA) carried out to explore the difference between genes and relationships with clinicopathological parameters.** The analysis of gene expression was made by PCA. On the dimension 1 (Dim 1), a clear discrimination can be noted between overexpressed genes (*AR*, *SRC3* and *EZH2*) and under expressed genes (*RAR beta 2*, *ER alpha, PGR* and *RGMA*). The overexpression of *AR*, *SRC3* and *EZH2* gene correlated with PSA level (PSA), clinical stage (patho) and Gleason score (Gleason).

Two separate epigenetic pathways have been shown to be dysregulated or impaired in cancer. The first is the well-known silencing of genes by DNA methylation and the second is the silencing of genes mediated by the Polycomb repressor complex 2, which is often independent of DNA methylation. Thus, to demonstrate the role of H3K27me3 mark on gene silencing, we measured *RAR beta 2, ER alpha*, *PRG*, *RGMA*, *SRC3*, *AR,* and *EZH2* mRNA expressions from prostate tissues using RT-qPCR. Figure 4 depicts the relative mRNA expression and statistical analysis showed significantly higher *EZH2*, *AR* and *SRC3* mRNA levels in patients with prostate cancer compared to normal patients. Contrary, *RAR beta 2*, *ER alpha*, *PGR,* and *RGMA* mRNA levels were decreased in prostate cancer tissue compared to normal one. Next, the analysis of gene expression was made by principal component analysis (PCA) to find a relationship with clinical pathological parameters. The first two principal components accounted for 68.31% of the total variance and explained the contrast between overexpressed genes (*AR*, *SRC3* and *EZH2*) and underexpressed genes (*RAR beta 2, ER alpha, PGR* and *RGMA*). Additional variables (Gleason score, PSA levels and clinical stages) positively correlated with the overexpression of *EZH2, SRC3,* and *AR* therefore with the under expression of *RAR beta 2, ER alpha, PRG* and *RGMA* (Figure 5). Using DFA analysis, only the variable Gleason score allowed to properly classify and predict patients belonging to different groups (GS ≤ 7 and GS > 7). On the other hand, PSA levels and clinical stages did not help establish the separation into different groups.

**Figure 6:**
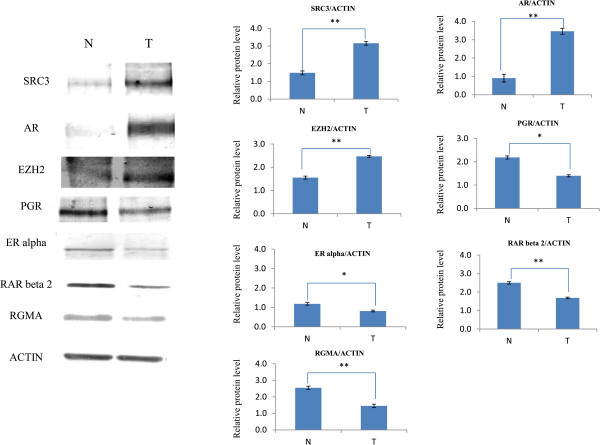
**EZH2 Expression is inversely correlated to RAR beta 2, ER alpha, PGR and RGMA expressions.** Protein expressions were analyzed using antibodies against SRC3 (155 KDa), AR (110 KDa), EZH2 (98 KDa), PGR (80 KDa), ER alphax (66KDa), RAR beta 2 (55 KDa) and RGMA (49 KDa). Anti-actin antibody (44 KDa) was used as the internal loading control. Representative data of 3 independent experiments is shown (left panel). Quantification of the western blot data is shown (right panel). The data is normalized to GAPDH (value = means ± SD, all results are statistically significant, *p < 0.05, **p < 0.001). The data is expressed as relative protein level. (N = Normal; T = Tumor).

To determine whether the gene expression data identified by mRNAs analysis resulted in biologically meaningful changes in protein expression, EZH2, RAR beta 2, ER alpha, PGR, RGMA, SRC3, and AR protein levels were assessed in prostate tissue samples using western blot analysis. As shown in Figure 6, the level of EZH2, SRC3 and AR proteins were higher in prostate cancer tissues with a Gleason score of at least 8. On the contrary, RAR beta 2, ER alpha, PGR, RGMA protein levels were notably reduced in prostate cancer tissues compared to normal tissues. These results are consistent with the mRNA expression levels data.

### Effect of DZNep and SAHA on cell viability

**Figure 7:**
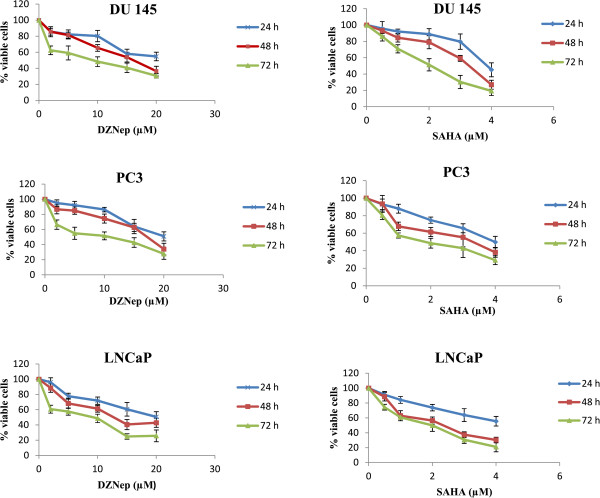
**Effects of DZNep and SAHA on cell viability.** DU145, PC3 and LNCaP cells were treated with DZNep (2–20 μM) and SAHA (0.5-4 μM) for 24 h, 48 h and 72 h. The percentage of viable cells was determined as the ratio between treated cells and control cells. The results were expressed as the mean of triplicate independent experiments. IC50 was calculated following formula: EXP(LN(conc > 50%)-((signal > 50%-50)/(signal > 50%-signal < 50%)XLN(conc > 50%/conc < 50%))).

To further determinate cell viability, we treated DU145, PC3 and LNCaP cells with 3-deazaneplanocin A (DZNep) and suberoylanilide hydroxamic acid (SAHA) using various concentrations for 24 h, 48 h and 72 h and then, the number of viable cells was counted. As shown in Figure 7, DZNep significantly decreased the cell proliferation in a concentration-dependent manner. DZNep displayed an IC50 value of 8.97 μM for DU145 against 10.76 μM for PC3 and 9.01 μM for LNCaP at 72 h of treatment. However, SAHA displayed an IC50 value of 2.05 μM for DU145, 1.88 μM for PC3 and 1.99 for LNCaP at 72 h of treatment. According to these results, 10 μM DZNep and 2 μM SAHA were chosen for mRNA experiments.

### Restoration of mRNA expression by DZNep and SAHA in prostate cancer cells

**Figure 8:**
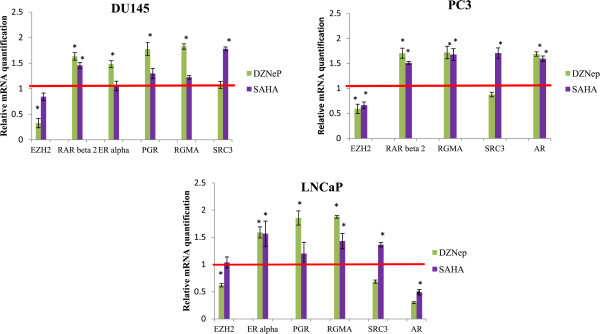
**DZNep and SAHA effects on prostate cancer cell lines.** Total RNAs were isolated from PC3, DU145 and LNCaP cells treated with 2 μM SAHA and 10 μM DZNep for 72 h. Results were analyzed by RT-qPCR. Relative changes in gene expressions compared to the control (the value of control was designed as 1) were calculated using comparative ΔΔCt method. The 18S RNA was used for normalization. Value = means ± SD from at least three measures, *p < 0.05.

To assess the effect of DZNep and SAHA, the levels of *RAR beta 2*, *ER alpha*, PRG, *RGMA*, *SRC3*, *AR* and *EZH2* mRNA were examined in prostate cancer cells using RT-qPCR. As shown in Figure 8, after 72 h of treatment, DZNep was able to decrease *EZH2* mRNA expression in LNCaP, PC3 and DU145 cells compared with control cells treated with DMSO 0.1% (p < 0.05). In the three cell lines, after 72 h, DZNep induced an increase of *RGMA* and *RAR beta 2* mRNA levels. This re-expression was associated with an *EZH2* decreased. In both AR-negative cell lines, only PC3 cells reactive *AR* expression after treatment with DZNep and SAHA. Any effect of DZNep on *SRC3* expression has been observed on prostate cancer cell lines. However, in LNCaP, DU145 and PC3 cells there was a moderate increase of *SRC3* expression after 72 h of treatment with SAHA. *ER alpha* and *PGR* expressions in DU145 and PC3 cells were undetected by RT-qPCR in control cells. However, in these two cell lines, *ER alpha* and *PGR* re-expressions were detected with DZNep or SAHA treatment (Figure 8).

## Discussion

The progression of prostate cancer, like other cancers, is facilitated by the epigenetic silencing of tumor-suppressor genes [21]. DNA hypermethylation in prostate cancer has been extensively studied [22, 23]. However, histone modification patterns have been found to predict the risk of prostate cancer [24].

The present study clearly pointed out H3K27me3 as an epigenetic mark involved in the silencing of *RAR beta 2, ER alpha*, *PGR,* and *RGMA* genes in prostate cancer. Similarly, one of our previous studies showed that H3K27me3 marks were also significantly higher in peri-tumoral tissues from prostate cancer patients compared to normal tissue. These results suggest that peri-tumoral tissues seem to present molecular features such as tumoral tissues and established H3K27me3 like an epigenetic mark pathogenically involved in neoplasia trough the silencing of genes [25]. Previous study has also demonstrated an increase of *RAR beta 2* and *Adenomatous polyposis coli* (*APC*) DNA methylation level in prostate tissue containing intraepithelial neoplasia and in adjacent non-neoplastic tissue [26].

The PCA analysis was used and enabled us to show the existing correlation between all the different analyzed variables. Additionally, the DFA analysis was used because different groups were known and also to help predict group membership. This enables to include many variables in the study in order to determine the ones that discriminated between groups. The results showed an evident correlation between H3K27me3 level and clinicopathological parameters in prostate cancer tissues. However, it should be clearly noted that, if the overall average of the variables is significantly different between cancer and normal tissue, it does not mean that this variable discriminates different assigned classes. Obviously, results showed that only Gleason score variable is the best predictor of the clinicopathological parameters which classify patients into each group according to H3K27me3 level.

The PSA levels and clinical stages allowed separating normal patients with prostate cancer patients, without distinguishing different classes of clinical stages and PSA levels. About the clinical stages, DFA analysis did not help discriminate T1 and T2 clinical stage groups. Moreover patients with high level of H3K27 marks do not necessarily have high PSA level.

To examine the correlation between the abundance of H3K27me3 and gene activity, mRNA transcript levels were quantified using RT-qPCR. Previous studies have identified EZH2 as upregulated in prostate cancer [27, 28]. Our results exhibited that, the group of patients with Gleason score >7 showed strong overexpression of *EZH2*, *AR,* and *SRC3* and eventually underexpression of *RAR beta 2, ER alpha, PRG,* and *RGMA* compared to normal patients group. The group with Gleason score ≤ 7 is intermediate between the two others. These results are in agreement with previous studies which showed an increase of *EZH2* in prostate cancer relatively to normal tissue. Similarly, the results demonstrated that AR was also increased in prostate cancer tissue. Indeed, *AR* is a potent oncogene which plays a crucial role in the early development of prostate cancer, as well as in metastatic castration-resistant progression in prostate cancer. Previous studies also showed that *AR* expression was tumor stage-dependent [29]. *SCR3* has also been identified as a key factor in the development of prostate cancer and there is an upregulation of *SRC3* in prostate cancer tissues. In fact, when AR was activated by hormones, AR bound to DNA in the nucleus cell and regulated gene expression through coactivators such as SRC3.

The identification of ER in the prostate gland was an indicator suggesting that locally produced testosterone metabolites with estrogenic activity may serve to balance the androgenic action in this tissue [30]. The data demonstrated a decrease in *ER alpha* expression in patients with Gleason score >7. This result indicated that, the estrogens via their ER alpha might inhibit growth of prostate cancer cells. Previously, we demonstrated that patients with Gleason score at 6 revealed no significant difference in the expression of *ER alpha* compared with the normal tissues [31]. However in this study, a positive correlation was observed between decreased *ER alpha* expression and tumor aggressiveness.

Unfortunately, very few studies have focused on the role of RGMA in prostate cancer. Li et al. (2012) observed that RGM proteins played inhibitor role in prostate cancer by suppressing cell growth, adhesion migration, and invasion [17]. In a comparative way, our studies showed that *RGMA* expression was down-regulated in most of the prostate cancer tissues and was positively correlated with Gleason score. On the other hand, another study showed similar findings in colon cancer [32].

Another key point is the fact that inactivation of tumor suppressor genes was a major contributing alteration factor in the initiation or progression of cancer. Like tumor suppressor genes, *RAR beta 2* was recurrently silenced in prostate cancer, predominantly by epigenetic mechanisms. Again, previous studies showed that promoter region of *RAR beta 2* was hypermethylated in more than 80% of human prostate cancer samples and prostate cancer cells leading to loss of *RAR beta 2* expression [15, 33]. A clear relationship was proven between decreased of *RAR beta 2* expression and high Gleason score late-stage clinical. Furthermore, our results showed high proportion of H3K27me3 marks at *RAR beta 2* promoter suggesting a strong involvement of polycomb group proteins in silencing of *RAR beta 2* gene in prostate cancer. Consequently, *RAR beta 2* may be silenced not only by DNA methylation as previously demonstrated [34].

To provide more evidence that these genes are H3K27me3-dependent silencing, we assessed the levels of *RAR beta 2*, *ER alpha*, *PGR,* and *RGMA* mRNA using RT-qPCR in PC3, DU145 and LNCaP cell lines after treatment with 3-Dezaneplanocin-A (DZNep), a potent pharmacologic inhibitor of EZH2. DZNep is an S-adenosyl-L-homocysteine hydrolase inhibitor which works across an indirect mechanism blocking S-adenosylmethinone-dependent methyltransferases by-product inhibition. Our results showed that DZNep induced a re-expression of *RAR beta 2* suggesting that DNA hypermethylation is not a predominant mechanism of silencing of *RAR beta 2*. It is also well known that the polycomb complex PRC2 recruits histone deacetylase proteins at the promoter region of target genes and subsequently mediates epigenetic transcriptional repression. However, treatment for prostate cancer cells with SAHA, a potent histone deacetylase (HDAC) inhibitor, results in depletion of *EZH2* and induction of *RAR beta 2* in prostate cancer cell lines. Other genes such as *PGR* and *ER alpha* were also studied because they have been identified in both normal and prostate cancer tissues. Few studies have reported the involvement of these two receptors in the prostate cancers [35]. But currently, neither mutation nor structural alterations of these receptor genes in prostate cancer have been reported to be responsible for *ER alpha* or *PGR* down-regulation. Hopefully, further studies in epigenetics will investigate this issue. However, it was known from previous studies, that the *ER alpha* promoter is extensively methylated both in prostate cancer cell lines and prostate cancer tissues, leading to *ER alpha* gene inactivation in prostate cancer [36]. Other studies have shown no significant difference between cancer tissues and normal prostate tissues [4]. We also evidenced a significant increase of H3K27me3 marks on *ER alpha* gene in prostate tumors compared to normal tissues. Our data added new mechanisms whereby histone methylation could silence *ER alpha* gene in prostate cancer. Besides, an increase of H3K27me3 marks on *PGR* gene can explain its downregulation in prostate cancer. These results have been confirmed by Western blotting in prostate tissues.

Our investigations have also shown an increase of H3K27me3 on *RGMA* gene which would explain the silencing of this gene in prostate cancer. According to the previous studies, results exhibited that, *RGMA* mRNA was decreased in the prostate cancer tissues compared to normal prostate tissues [10]. However, the mechanisms involved in this regulation of prostate cancer cells are still unknown. In PC3, DU145 and LNCaP cell lines, we found reactivation of *RGMA* expression after DZNep treatment supporting the hypothesis that *RGMA* is silenced by EZH2. In order to compare DZNep to other epigenetics drugs such as SAHA, we also observed reactivation of *RGMA* expression with SAHA. Surely, HDACs bind PRC2 complex leading to a series of reactions, including decreasing acetylation of H3K27, favoring its methylation and inhibiting gene expression. SAHA would be expected to counteract this activity, resulting in increased acetylation and gene expression.

It should be noted that H3K27me3 would have no effect on *SRC3* expression. Using ChIP-qPCR, there is no significant discrepancy between the cancerous group and the normal group. However, *SRC3* was overexpressed in prostate cancer compared to normal tissues. When we treated prostate cancer cells with SAHA, it was observed an increase of *SRC3* expression which is a histone acetyltransferase activity [4].

The AR signalling pathway is a key factor in the development and progression of prostate cancer. DZNep reduces the transcriptional activity of *AR* in LNCaP cells, suggesting that DZNep negatively regulated *AR* expression. Furthermore, the initiation of prostate cancer has been linked to activation of AKT pathway due to loss or mutation of PTEN. Additionally over-expression of *AR* and *EZH2* appeared to be important to promote the progression of prostate cancer. The underlying mechanism remained unknown. Previous studies have shown that, overexpression of *AR* alone led to a repression of *EZH2* expression while knockdown of *AR* increased *EZH2* expression in LNCaP cells [37]. This may be due to activation of AKT promoting EZH2 phosphorylation at Serine 21, which down-regulates its methyltransferase activity by blocking EZH2 binding to histone H3, decreasing H3K27me3 marks leading to de-repressing epigenetic silencing [38].

Moreover, previous studies reported that, SAHA was more efficient in terms of growth inhibition and induction of cell death in androgen-responsive cells [39], suggesting that a component of the activity of SAHA in prostate cancer cells relates to the presence of a functional androgen signalling axis and that HDAC inhibitors decreased AR protein levels without significantly affecting AR protein stability [40, 41]. Conversely, in DU145 and PC3 cells, SAHA led to a re-expression of *AR*. Also, there was no significant difference of SAHA activity over *EZH2* expression.

This data suggested that for some genes, the silencing by EZH2 could be an alternative strategy to override to DNA methylation. Certainly, *EZH2* was overexpressed in prostate cancer and played a crucial role in several steps of the metastatic process as described in previous studies.

## Methods

### Biopsy collection

The 137 men were hospitalized at the Clermont-Ferrand University Medical Center (France) between 2012 and 2013, and had 12 sextant biopsies [42]. With a letter of consent, patients accepted to give a sample for research. This procedure corresponded to collection of biological samples declared to "Le Ministère de l’Enseignement Supérieur et de la Recherche" with registered number: DC-2008-558. The anatomo-pathological examination diagnosed the stage of cancer development. Each biopsy was stored in a cryotube containing nitrogen solution at −196°C at the Jean Perrin Center tumorbank, biological resource center (CRB), accredited under No. AC-2013-1882. The clinicopathological characteristics of all the analyzed prostate tissues are listed in Table 4 and Table 5.

**Table 4:** **Summary of clinicopathological parameters in prostate samples for ChIP-qPCR**

CASES	TT	NT
Total cases (n = 65)	32	33
Age at diagnosis (years)		
<49	0	1
50-59	6	10
60-69	17	17
>70	9	5
PSA baseline (ng/mL)		
<4	0	4
4-10	14	23
10-20	8	4
>20	10	2
Clinical stage		
T1c	17	-
T2a	7	-
T2b	6	-
T2c	1	-
T3	1	-
Gleason score		
≤7	19	-
>7	13	-

TT: tumoral tissues; NT: normal tissues; PSA: prostate-specific antigen.

**Table 5:** **Summary of clinicopathological parameters in prostate samples for RT-qPCR**

CASES	TT	NT
Total cases (n = 72)	34	38
Age at diagnosis (years)		
<49	0	0
50-59	2	10
60-69	11	21
>70	21	7
PSA baseline (ng/mL)		
<4	1	2
4-10	14	25
10-20	9	11
>20	10	0
Clinical stage		
T1c	13	-
T2a	2	-
T2b	10	-
T2c	2	-
T3	6	-
T4	1	-
Gleason score		
≤7	19	-
>7	15	-

TT: tumoral tissues; NT: normal tissues; PSA: prostate-specific antigen.

### Cell lines and culture conditions

Three human prostate cancer cell lines, including the DU145, PC3, and LNCaP obtained from american type culture collection (ATCC, Manassas, VA, USA) were used. The cells were cultivated in RPMI 1640 medium for LNCaP, in F-12 K medium for PC3, and in eagle’s minimum essential medium (EMEM) for DU145 (Gibco, Grand Island, NY, USA). All cultures were supplemented with 10% heat-inactivated fetal bovine serum (FBS) (Life Technologies, Carlsbad, CA, USA), 1% glutamin, and 100 U/ml gentamicin (Sigma-Aldrich, St Louis, MO, USA). Cells were maintained as monolayers in an incubator within humidified atmosphere of 95% air and 5% CO_2_ at 37°C.

### Chemical treatments

To determine the optimal concentration of 3-deazaneplanocin A (DZNep) and suberoylanilide hydroxamic acid (SAHA) (Sigma-Aldrich St. Louis, MO, USA) in prostate cancer cell lines, DZNep and SAHA were dissolved in dimethyl sulphoxide (DMSO, Sigma-Aldrich, St Louis, MO, USA). Final DMSO concentration did not exceeded 0.1%. The same concentration of DMSO was used as a control for these experiments. We measured cell viability using a Scepter™ 2.0 Cell Counter (Millipore, Billerica, MA, USA) in accordance with the manufacturer’s standard operating procedures. To identify the demethylating and deacetylating effects of DZNep and SAHA, DU145, LNCaP and PC3 cells were distributed in six-well culture plates at a density of 0.5×10^5^ cells per well. After 24 hours, cells were treated with increased doses of DZNep and SAHA using 10 μl DMSO for respectively and gradually 24, 48 and 72 hours. After treatments, cells were washed with phosphate buffered saline (PBS, Life Technologies, Carlsbad, CA, USA), trypsinized, harvested and the number of viable cells was counted. Viable cells were presented as a percentage of the untreated cells control and IC_50_ is done by linear interpolation between concentrations just above and beneath 50% inhibition in the response dose curve.

### Chromatin extraction and shearing

Chromatin extraction was carried out on carcinoma and normal prostate samples (Table 4). Tissues were treated with 1% formaldehyde for 15 min to crosslink histone to DNA. The crosslinking reaction was stopped with 0.125 M fresh glycine for 5 min. After washing by cold PBS, the samples were grinded with TissueRuptor® (Qiagen, Hilden, Germany) until getting a homogeneous suspension. The lysate was centrifuged at 4000 g for 10 min at 4°C and the cell pellet was re-suspended in lysis buffer (5 mM PIPES pH 8; 85 mM KCL; 0.5% IGEPAL, protease inhibitor cocktail) and incubated in ice for 15 min. Then, nuclei were centrifuged at 4000 g for 10 min and the pellet was re-suspended in shearing buffer (Diagenode, Seraing, Belgium), incubated in ice for 5 min and sonicated (Bioruptor™ sonicator, Diagenode) for 40 min. This produced chromatin fragments of 100 to 600 bp suitable for ChIP assays as they cover 2 to 3 nucleosomes. Immediately after sonication, the samples were cleaned by centrifugation at 4000 g for 10 min at 4°C and supernatants containing the sheared chromatin were transferred into new tubes. Sonicated chromatin was used for DNA extraction (sonication optimization experiments; data not shown) or for chromatin immunoprecipitation (ChIP) assay.

### Chromatin immunoprecipitation

ChIP was performed using auto ChIP kit (Diagenode) according to manufacturer’s instructions. Anti-human H3K27me3 (pAb-069-050, Diagenode) produced in rabbit and also non-immune rabbit IgG (negative control) (kch-504-250, Diagenode) were used. The ChIP was carried out by SX-8X® Automated System (Diagenode) according to the protocol provided by the manufacturer. The reaction was incubated for 2 h for antibody coating with protein A-coated magnetic beads, then for 10 h at 4°C for immunoprecipitation reaction. Later on, 1 μL of proteinase K was added and the reverse cross-linking was performed for 45 min at 65°C. The immunoprecipitated DNA and input samples were analyzed by real-time qPCR.

### Quantitative real-time PCR

Real-time PCR was performed in triplicate on a 25 μl reaction containing 1X TaqMan Universal PCR Master Mix (Applied Biosystems) and 400 nM of each of forward and reverse primers (Sigma-Aldrich), 250 nM of probe (Sigma-Aldrich) and 4.25 μL of water. TaqMan qPCRs were then carried out for *EZH2, RAR beta 2*, *ER alpha, PGR, RGMA,* and *SRC3* genes. The oligonucleotide primers and probes are shown in Table 6. The efficiency of chromatin immunoprecipitation of particular genomic locus was calculated from qPCR data and reported as a percentage of starting material: % (ChIP/total input).

**Table 6:** **Forward, reverse primers and probes used for ChIP-qPCR for amplification of the gene promoter region**

Genes	qPCR primer sequences	qPCR MGB Probes (Taqman®)
*EZH2*	Forward AGTGCAATGGCGCGATCT	TCACCGCAACCTC
	Reverse GAGGCATGAGAATCGCTTGAA	
*RAR beta 2*	Forward GCACGTAGGCTGTTGGTCTTT	CCAGCCCCGAATC
	Reversre GCTGGCTTGTCTGTCATAATTCA	
*PGR*	Forward GAGCCGCGTGTCACTAAATTG	CGTCGCAGCCGCA
	Reverse TCACAAGTCCGGCACTTGAG	
*ER alpha*	Forward CCCTGACATTGGCTTAAACATCA	TCCAGGCACAACTC
	Reverse TCTTTGGGATCGCTCCAAAT	
*RGMA*	Forward CTGCCAGGTCGGGAGTGT	AGAGGAGCAAGTTTG
	Reverse CACAGCCATAGGGCCTTCTC	
*SRC3*	Forward AAAATTAAGGGCAGGGCTAGGA	TCCGGATCCCGAGGGAGCTCC
	Reverse GTGCGGCCGCTTTCG	



Ct (input) and Ct (ChIP) are threshold values obtained from exponential phase of qPCR for the immunoprecipated DNA sample and input sample respectively. log(X%)/log2 accounted for the dilution 1/X of the input.

### Reverse transcription and qPCR

One microgram of mRNA from each sample was reversely transcribed in a total volume of 15 μl using first strand cDNA synthesis kit (GE Healthcare Life Science, Piscataway, NJ, USA) according to the manufacturer’s instructions. The resulting cDNA was then quantified with the TaqMan method (ABI Prism 7900 HT, Applied Biosystems). Duplex PCR was carried out in 96-well plate using 10 ng of cDNA in a total volume of 25 μl containing 12.5 μl TaqMan Gene Expression Master Mix (2X) (4369016, Applied Biosystem), 200nM of *EZH2*, *RAR beta2*, *ER alpha*, *PGR*, *RGMA*, *SRC3*, and *AR* Applied Biosystems assays-on-demand: *EZH2* (Hs01016789_m1), *RARbeta2* (Hs00977140_m1), *ER alpha* (Hs00174860_m1), *PGR* (Hs01556702_m1), *RGMA* (Hs00297192_m1), *SRC3* (Hs01105251_m1) and *AR* (Hs00171172_m1), 10 μM 18*S* rRNA primers and 5 μM 18*S* rRNA TaqMan probe. 18*S* primers and TaqMan probe were purchased as follows: forward: 5’-CGG CTA CCA CAT CCA AGG AA-3’, reverse: 5’-GCT GGA ATT ACC GCG GCT-3’, probe: 5’-TGC TGG CAC CAG ACT TGC CCT C-3’ (VIC). Data were collected using an AB Prism 7900 Sequence Detector System (Applied Biosystem) for 45 cycles (50°C during 2 min, 95°C for 10 min, 95°C for 15 s and 60°C for 1 min). Samples were normalized to 18*S* rRNA level. The comparative cycle threshold (CT) method (2^-ΔΔCt^) was used to calculate the relative gene expression. All data were generated at least in triplicate and expressed as mean ± SD. Genes were considered significantly expressed and their transcript measurable if their corresponding Ct value was less than or equal to 35.

### Western-blotting

Total protein extractions were performed using RIPA buffer (Sigma-Aldrich) according to the manufacturer’s instructions. Protein concentration was assayed using the Bradford method (Bio-Rad, Hercules, CA, USA) and equal amount of proteins (25 μg) was separated by electrophoresis on 4-12% SDS-PAGE gels (Bio-Rad Laboratories,Hercules, CA, USA) and transferred to polyvinylidene fluoride membranes (GE healthcare). Membranes were blocked with 5% nonfat milk in 0.1% TBS- tween for 1 hour at room temperature, washed, and incubated overnight at 4°C with primary antibodies in 1% nonfat milk and 0.1% TBS-tween. Primary antibodies used were as follow: anti-EZH2 (1:500, pAb-039-050, Diagenode), anti-RAR beta 2 (1:500, GTX12011, GeneTex), anti-ER alpha (1:250, AC-066-100, Diagenode), anti-PGR (1:250, PAB12723, Abnova), anti-RGMA (1:250, H00056963-M01, Abnova), anti-SRC3 (1:750, MAB7999, Abnova, Walnut, CA, USA), anti-AR (1:1000, GTX73078, GeneTex, Irvine, CA, USA) and anti-β Actin (1 :4000, CP01, Millipore). A secondary antibody consisted of anti-mouse IgG or anti-rabbit IgG alkaline phosphatase conjugates (1:2000, S372B and S373B respectively, Promega, Madison, WI, USA). After final washes, blots were developed using Western Blue Stabilized substrate for alkaline phosphate (2016-08-26, Promega).

### Statistical analysis

#### Analysis of variance

All statistical analysis were performed using R 3.0.1 software [43] and the statistical packages agricolae [44], HH [45] and multcomp [46]. All the data obtained were statistically analyzed with a one-way ANOVA to test the level of statistical significance of different groups on H3K27me3 levels and relationships between clinicalpathological parameters on different genes. Post-hoc procedures were used when the F-test was significant (p < 0.05). Multiple comparisons among means were examined by a Tukey’s Post Hoc test. The level of statistical significance was set at p < 0.05.

#### Principal Component Analysis

The major aim of Principal Component Analysis (PCA) is the orderly simplification of a large number of inter-correlated measures to a few uncorrelated representative constructs or factors [47]. In this study, a small number of linear combinations (called principal components) are derived from a set of variables (genes) measured on several observations (patients) that retain as much as possible of the information about the original variables. This goal typically overrides in empirical research its secondary aim, which is interpretation of the principal components. To a large extent, the interpretation of principal components is generally guided by the degree to which each variable is associated with a particular component [47]. The main outputs of the principal component analysis are the variances of the principal components and two plots: the projections of the original variables; and the observations onto the plane made by the two first principal components. In the first plot, as all variables are normalized (mean equal to zero and variance equal to 1), the distances between the projections of the variables and the centre are equal to 1, meaning that the projections near the circumference of the circle belong to the plane of the two first principal components, and the projections near the centre do not belong to the plane as they appear to be orthogonal. Those variables, found to be most closely related to a component in question are used as a guide for its interpretation. Principal Component Analysis was performed using the statistical package FactoMineR [48].

#### Discriminant Analysis

Discriminant analysis is a powerful descriptive and classificatory technique to first describe characteristics that are specific to distinct groups (called descriptive discriminant analysis) and secondly classify cases (i.e., patients, subjects, participants) into pre-existing groups based on similarities between different cases that belong to a specific group [49]. Discriminant analysis can answer some questions that include: (a) in which ways do various groups in a study differ? (b) What differences exist between and among the number of groups on a specific set of variables? (c) Which continuous variables best characterize each group, or, which continuous variables are not characteristic of the individual groups? (d) Given the results of a multivariate analysis of variance indicating that group differences exist in the data, what specific variables best account for these differences? [49]. Given two or more groups or populations and a set of associated variables one often wants to locate a subset of the variables and associated functions of the subset that leads to maximum separation among the centroids of the groups. The goals of a discriminant analysis are about constructing a set of discriminants that may be used to describe or characterize group separation based upon a reduced set of variables, to analyze the contribution of the original variables to the separation, and to evaluate the degree of separation. To test the contribution of each variable, Wilks' lambda is used in an ANOVA [48] test of mean differences in discriminant analysis, so that the smaller the lambda for an independent variable, the more that variable contributes to the discriminant function. Lambda varies from 0 to 1, with 0 representing group means differ and 1 meaning that all group means are the same. The F test of Wilks' lambda shows which variables contributions are significant. Discriminant Analysis was performed using the statistical package rrcov [50].

## Conclusion

Based on the overall results, we believe in a direct role of EZH2 in silencing of *RAR beta 2*, *ER alpha*, *PGR,* and *RGMA* genes via H3K27me3 mark in prostate cancer and therefore indicates adverse prognosis. Treatment with DZNep led to the reactivation of genes silenced by epigenetic mechanisms in prostate cancer. In view of these results, we demonstrated that histone modifications contributed to the onset and progression of prostate cancer and H3K27me3 would be considered as a promising biomarker. The identification of these molecular markers will be used to supplement clinical markers and will allow us to better predict cancer progression.
